# First record of *Oscheius myriophilus* (Poinar, 1986) (Rhabditida: Rhabditidae) from Iran; and its efficacy against two economic forest trees pests, *Cydalima perspectalis* (Walker, 1859) (Lepidoptera: Crambidae) and *Hyphantria cunea* (Drury, 1773) (Lepidoptera: Erebidae) in laboratory condition

**DOI:** 10.21307/jofnem-2021-035

**Published:** 2021-04-01

**Authors:** Reihaneh Gholami Ghavamabad, Ali Asghar Talebi, Mohammad Mehrabadi, Mohammad Ebrahim Farashiani, Majid Pedram

**Affiliations:** 1 Department of Agricultural Entomology, Faculty of Agriculture, Tarbiat Modares University, P.O. Box 14115-336, Tehran, Iran; Research Institute of Forests and Rangelands, Agricultural Research, Education and Extension Organization (AREEO), Tehran, Iran; Department of Plant Pathology, Faculty of Agriculture, Tarbiat Modares University, P.O. Box 14115-336, Tehran, Iran

**Keywords:** Box tree moth, Entomopathogenic nematode, Fall webworm, Forest pests, Gilan, Pathogenicity, Phylogenetic

## Abstract

The box tree moth (BTM), *Cydalima perspectalis* and the fall webworm (FWW), *Hyphantria cunea* are two invasive pests of forest trees that have been recorded from Hyracinan forests in north Iran for the first time in 2016 and 2002, respectively. In a search for tentative native entomopathogenic nematode species (EPNs) with potential biocontrol ability against lepidopteran pests of forest trees in north Iran, *Oscheius myriophilus* was isolated by soil-baiting method from forests of Amlash in the east of Gilan province. The Iranian isolate of this species is characterized by 870–1,247 μm long hermaphrodites having 17–20 μm long stoma, vulva at 45.5–53.1% of body length, tail 90–126 μm long, common males with 38–49 μm long spicules and 583–791 μm long dauer larvae. Compared to the type and other populations, no remarkable differences were observed for this population. The phylogenetic affinities of this isolate with other rhabditid nematodes were studied using partial sequences of small, and the D2-D3 expansion segments of the large subunit ribosomal DNA (SSU and D2-D3 LSU rDNA). This is the first record of the species from Iran. The pathogenicity of Iranian isolate of *O. myriophilus* was evaluated on the larvae of two important aforementioned forest trees pests, BTM and FWW under laboratory conditions. The results indicated that the suspension of 500 infective juveniles per ml (IJs/ml) of the nematode was the most effective treatment on fifth instar larvae of BTM and FWW, causing 100 and 95% mortality after 48 h, respectively. The lethal concentration 50 (LC50) values of the nematode were 74.5, 152.7, 99.9, and 197.3 IJs/ml on fifth and fourth instar larvae of BTM, and fifth and fourth instar larvae of FWW, respectively, after 48 h at 25°C and 60% relative humidity. Together, present results corroborated the efficacy of the Iranian isolate of *Oscheius myriophilus* for biocontrolling of BTM and FWW in laboratory conditions.

The Hyrcanian (Caspian) forests are natural environments in the north Iran that are known as the ecological biodiversity hotspots. So far, 3,234 species belonging to 856 genera and 148 families of vascular plants have been reported from this region (Akhani et al., 2010). The box tree moth (BTM), *Cydalima perspectalis* (Walker, 1859) (Lepidoptera: Crambidae), is a native pest in the subtropical regions of eastern Asia (Bras et al., 2019). It was detected in 2007 in southwestern Germany (Billen, 2007) and the Netherlands (Muus et al., 2009), and in recent years invaded other European countries (Bras et al., 2015, 2016). In Iran, it is an invasive and serious pest of *Buxus* L. trees, which was first detected in Banafsheh forests Park, Chalus in 2016; and it is now distributed all over the Hyrcanian forests in the north Iran (Ahangaran, 2016). The damage caused by the larvae of this pest in the foliage, often leads to a complete defoliation of the trees (Wan et al., 2014). The fall webworm (FWW), *Hyphantria cunea* (Drury, 1773) (Lepidoptera: Erebidae) is another invasive pest in Hyrcanian forests of Iran. The origin of this moth is North America that has become an invasive pest in Europe and Asia. In Iran, it was recorded for the first time from Gilan province in 2002 (Rezaei et al., 2004). The FWW is a polyphagous insect that feeds on the leaves of wide range of forest trees, orchards, cultivated crops, and herbaceous plants (Schowalter and Ring, 2017; Rezaei et al., 2006). According to the guide to the biological control of pests in forests by Food and Agriculture Organization of the United Nations (FAO Forestry) by Kenis et al. (2019), forest pests can be managed by deployment of resistant or tolerant germplasms and also using chemical, cultural, and biological methods in an integrated pest management (IPM) program. The first classical biological control project against forest insects using parasitoids and predators was carried out in Europe, Japan, North Africa, and Asia in 1905 (Doane and McManus, 1981). In a biological control of pests using nematodes, the mass production and release of *Deladenus siricidicola* Bedding, 1968 (Neotylenchidae) for biological control of *Sirex noctilio* Fabricius, 1793 (Hymenoptera: Siricidae) was performed in Australian forests (Bedding and Akhurst, 1974). Park et al. (2012) showed that *Plutella xylostella* L. larvae were most susceptible to *Rhabditis blumi* Sudhaus, 1974 showing a 88.0% rate of mortality. The application technology for use of EPNs in biological controlling programs was reviewed by Shapiro-Ilan et al. (2006). Also, application of EPNs for biocontrol of insect pests at above and below ground; and their commercialization were reviewed by Lacey and Georgis (2012). The family Rhabditidae (Örley, 1880) is one of the richest groups of free-living nematodes that are found in all kinds of habitats including decaying bacteria-rich substances such as decaying plants and cadavers or are associated with different animals, especially the insects (Andrássy, 1983). Sudhaus and Fitch (2001) and Sudhaus and Schulte (1989) reviewed the systematics, phylogeny, ecology, and biology of the genera of Rhabditidae. Based on a recent analysis by Sudhaus (2011), 38 genera are gathered under the family Rhabditidae, harboring 368 valid species. A pictorial key to the 38 valid genera of Rhabditidae was provided by Scholze and Sudhaus (2011). The genus *Oscheius* Andrássy, 1976 is delimited by the characters of the lip region and stoma, tail shape, male posterior body region and bursa, spicules and gubernaculum (Scholze and Sudhaus, 2011). It belongs to the family Rhabditidae, has two intragenus groupings, namely Dolichura and Insectivora (Sudhaus, 1976), the first group being characterized by having a peloderan bursa, thin tubular spicule tips; and the second group is characterized by a leptoderan or pseudopeloderan bursa, and crochet needle-shaped spicules. Currently, there are 23 species of Insectivora group and 16 species of Dolichura group (Abolafia and Peña-Santiago, 2019). Some species of *Oscheius* are necromenic (Sudhaus and Schulte,1989; Dillman et al., 2012). In recent studies, evidences have corroborated the pathogenicity of *Oscheius* spp. on insects, a behavior of which mainly correlated with the endosymbiont bacteria belonging to two genera *Serratia* Bizio, 1823 and *Enterococcus* (ex Thiercelin and Jouhaud, 1903( e.g. *Oscheius rugaoensis* Zhang et al., 2012 (Zhang et al., 2012), *O. chongmingensis* Zhang et al., 2008 (Fu and Liu, 2019; Liu et al., 2012), *O. myriophilus* (Liu et al., 2016; Castro-Ortega et al., 2020), *O. microvilli* Zhou et al., 2017 (Zhou et al., 2017), *O. safricana* Serepa-Dlamini and Gray, 2018 (Serepa-Dlamini and Gray, 2018) and *O. basothovii* Lephoto and Gray, 2019 (Lephoto et al., 2015; Lephoto and Gray, 2019). The bacterial symbiont is the important and often overlooked determinant of success of an entomopathogenic nematode (Labaude and Griffin, 2018). *O. myriophilus* was described as an associate of the garden millipede, *Oxidis gracilis* (Koch) (Diplopoda: Paradoxosomatidae) in California by Poinar (1986). It was later isolated from millipedes in South Australia by Sudhaus and Schulte (1989), European mole cricket, *Gryllotalpa gryllotalpa* L. from the Black Sea region of Turkey (Erbaş et al., 2017) and a sugar cane crop soil, in Mexico (Castro-Ortega et al., 2020). Recent studies by Liu et al. (2016) have shown that *O. myriophilus* is an entomopathogenic nematode; being associated with the bacterium *Serratia nematodiphila* Zhang et al., 2009. Castro-Ortega et al. (2020) isolated the bacterial colonies *Serratia marcescens* Bizio, 1823 from *Oscheius myriophilus,* the bacterium which causes a 100% mortality of the 5th instar larvae of *Galleria mellonella* L. in 1 × 10^5^ concentration. According to them, *Oscheius myriophilus* has the potential to be used in the future as a biological control agent in Mexico. Fu and Liu (2019) correlated the pathogenicity of dauer juveniles of *O. chongmingensis* with the bacterial genera *Bacillus* Cohn, 1872, *Albidiferax* Ramana and Sasikala, 2009, *Acinetobacter* Brisou and Prévot, 1954 and *Rhodococcus* Zopf, 1981, isolated from the gut of the nematode. There have been few studies on identification, ecology and biology of *Oscheuis* spp. in Iran. Hassani et al. (2012) reported *O. tipulae* Lam and Webster, 1971 from Iran in the shape of an abstract. *Oscheuis rugaoensis* was reported from Iran, isolated from the white grub larvae *Polyphylla adspersa* Motschulsky, 1853 (Coleopera: Scarabaeidae) (Darsouei et al., 2014). Valizadeh et al. (2017) recovered three species *O. necromenus* (Sudhaus and Schulte, 1989), *O. onirici* Torrini et al., 2015 and *O. tipulae* in association with bark beetle galleries in Tehran, Iran. The application of native EPNs is preferred for more successful and environmental low-risk purposes (Labaude and Griffin, 2018). The recovery of *O. myriophilus* in present study from north Iran provided a chance for examining its potential biocontrol ability on two devastating forest tree pests dominating north Iran.

Thus, the objectives of this study were to (i) characterize the Iranian isolate of *O. myriophilus* recovered from north Iran based upon traditional and molecular criteria, and (ii) assess its biocontrol ability against the larvae of two invasive forest pests, BTM and FWW, in laboratory condition.

## Materials and methods

### Nematode isolation, culture, and morphological observation

*Oscheius myriophilus* was collected from forests of Amlash (37°08’N, 50°16’E), Gilan province, north Iran, in May 2019 using the baiting technique (Bedding and Akhurst, 1975) and the last instar larvae of *Galleria mellonella*. No other nematodes were collected using this method in this experiment. The dead insect larvae were transferred to Petri dishes with filter paper and covered with plastic according to white trap method (White, 1927). Dauer/infective larvae were emerged and stored in distilled water at 8°C (Kaya and Stock, 1997). In all, 20 last instar larvae of *G. mellonella* were exposed to 2,000 infective juveniles of the nematode in a Petri dish (60 × 15 mm) plates lined with moistened filter papers at 20°C (Nguyen and Hunt, 2007). The male and hermaphrodite nematodes were obtained by dissecting *G. mellonella* larvae after 3–4 days of the infection with infective larvae in 1% saline (NaCl) solution. The infective larvae were appeared after one week of infection. Adults and infective larvae of nematode were fixed and transferred to glycerin for light microscopic studies according to De Grisse (1969). Photographs were taken using an Olympus DP72 digital camera attached to an Olympus BX51 microscope equipped with differential interference contrast (DIC). Specimens were measured by a drawing tube attached to a Nikon Eclipse E600 light microscope. The voucher specimens of the nematode were deposited in Nematology Laboratory of Tarbiat Modares University, Tehran, Iran. Morphological characters and morphometric data of the type population (Poinar, 1986) and other populations reported under the species name (see Table 1) were used for morphological comparisons.

**Table 1.** Morphometrics of *Oscheius myriophilus* (Poinar, 1986) recovered from Gilan province, north Iran, and comparison with data of the type population, the population from Australia and the population from Turkey

	This study			Type population studied by Poinar (1986)			Sudhaus and Schulte (1989)		Erbaş et al. (2017)
Locality	Iran			California			Australia		Turkey
Host insect	*Galleria mellonella*			*Oxidis gracilis* (Koch)			*O. gracilis*		*Gryllotalpa gryllotalpa* (L.)
Character	Male	Hermaphrodite	Infective larvae	Male	Hermaphrodite	Infective larvae	Male	Hermaphrodite	Infective larvae
*n*	20	20	20	10	10	6	–	–	–
L	1,002.1 ± 61.3	1,020 ± 129.6	672.3 ± 71.4	1,270	1,320	564	–	–	630.2 ± 31.5
	(867–1,053)	(862–1,247)	(583–791)	(830–1,470)	(1,200–1,500)	(504–611)	(841–1,175)	(792–1,530)	(571.3–693.9)
a	15.3 ± 2.1	19.6 ± 2.1	24.2 ± 1.8	–	–	–	–	–	25 ± 2.2
	(13.2–18.7)	(17.0–22.7)	(21.0–27.9)	(18.4–21.9)	(19.1–21.3)	–	–	–	(20.5–28.5)
b	5.1 ± 0.2	5.8 ± 0.5	4.9 ± 0.3			–	–	–	4.7 ± 0.2
	(4.8–5.5)	(5.0–6.9)	(4.6–5.7)	(5.16–7.36)	(6.8–7.7)	–	(4.0–6.1)	(4.2–8.5)	(4.3–5.2)
c	18.1 ± 0.9	9.4 ± 0.9	7.1 ± 0.4			–	–	–	9.3 ± 0.9
	(16.6–19.2)	(8.0–10.9)	(6.6–7.9)	(14.8–20.4)	(2.2–3.5)	–	(14.4–21.8)	(8.9–13.0)	(8.3–11.6)
c'	1.7 ± 0.07	5.0 ± 0.5	5.5 ± 0.3	–	–	–	–	–	–
	(1.5–1.8)	(4.1–5.7)	(5.1–6.6)	–	–	–	–	(3–4)	–
V%= (distance from anterior end to vulva/L)×100	–	49.9 ± 2.0	–	–	–	–	–	–	–
	–	(45.5–53.1)	–	–	–	–	–	–	–
Stoma length	17.5 ± 0.5	17.6 ± 0.8	16.1 ± 1.4	17	20	–	–	–	–
	(17–18)	(17–20)	(14–20)	(16–19)	(18–21)	–	–	(18–21)	–
Stoma width	3.4 ± 0.5	4.2 ± 0.2	2.7 ± 0.3	3.2	3.2	–	–	–	–
	(3–4)	(4–4.5)	(2.5–4)	–	–	–	–	<4.5	–
Pharynx length	196 ± 9.7	176.3 ± 17.1	134.5 ± 7.1	187	185	129	–	–	134.6 ± 3.2
	(180–208)	(144–200)	(125–145)	(161–200)	(174–193)	(120–136)	–	–	(128.8–139.8)
Max. body diam.	66.8 ± 11.5	52.3 ± 6.8	27.8 ± 3.4	63	62	23	–	–	25.4 ± 2.7
	(47–79)	(42–66)	(24–33)	(38–80)	(57–70)	(19–26)	(52–72)	(52–108)	(21.0–30.4)
E pore from anterior end	165.9 ± 11.1	149.3 ± 9.3	116.3 ± 4.6	198	179	107	–	–	108 ± 6.7
	(142–177)	(135–167)	(110–129)	(149–229)	(165–190)	(97–114)	–	–	(97.8–118.8)
Nerve ring from anterior end	126 ± 4.7	121.1 ± 9.7	94.5 ± 6.7	150	139	89	–	–	110.5 ± 4.9
	(118–130)	(105–135)	(83–105)	(117–165)	(126–146)	(83–96)	–	–	(100–116.8)
Rectum length	–	63.7 ± 4.8	–	–	79	–	–	–	–
	–	(55–80)	–	–	(56–96)	–	–	–	–
Anal body diam.	32.3 ± 1.8	21.7 ± 2.4	16.9 ± 0.9	39	25	15	–	32	12.3 ± 1
	(30–35)	(17–26)	(15–18)	(32–45)	(22–28)	(14–16)	–	–	(10.4–13.8)
Tail	55.3 ± 3.3	108.1 ± 9.8	93.7 ± 5.0	65	117	78	–	–	82.1 ± 6.2
	(52–60)	(90–126)	(85–100)	(56–72)	(108–135)	(75–80)	–	–	(72.2–92.2)
Ratio of tail length to rectum	–	1.7 ± 0.1	–	–	–	–	–	1.5	–
	–	(1.1–1.8)	–	–	–	–	–	(1.4–2.3)	–
Spicules length	44.5 ± 3.8	–	–	47	–	–	–	–	–
	(38–49)	–	–	(32–54)	–	–	(27–39)	–	–
Spicules width	7.1 ± 0.3	–	–	10	–	–	–	–	–
	(7–8)	–	–	(8–11)	–	–	–	–	–
Gubernaculum length	20.0 ± 1.0	–	–	28	–	–	–	–	–
	(18–21)	–	–	(19–32)	–	–	–	–	–
Gubernaculum width	0.9 ± 0.09	–	–	0.87	–	–	–	–	–
	(0.8–1.1)	–	–	(0.64–1.20)	–	–	–	–	–
D%= (E pore from anterior end/Pharynx length) ×100	84.6 ± 3.7	85.1 ± 6.8	87.1 ± 3.5	–	–	–	–	–	–
	(78.8–90.5)	(76.3–100.0)	(82.5–94.4)	–	–	–	–	–	–
E%= (E pore from anterior end/Tail) ×100	300.2 ± 16.8	138.7 ± 10.2	125.5 ± 5.0	–	–	–	–	–	–
	(273–323)	(124.3–160.0)	(118.0–136.3)	–	–	–	–	–	–
SW%= (Spicules length/Anal body diam.) ×100	137.8 ± 8.2	–	–	–	–	–	–	–	–
	(126.6–150.0)	–	–	–	–	–	–	–	–
GS%=(Gubernaculum length/Spicules length)×100	45.1 ± 2.5	–	–	–	–	–	–	–	–
	(41.6–48.8)	–	–	–	–	–	–	–	–

### Molecular methods and phylogenetic analyses

For molecular studies, a single live nematode specimen was transferred to 20 μl of TE buffer (10 mM Tris-Cl, 0.5 mM EDTA, pH 9.0; Qiagen) on a clean slide, heat killed, photographed and squashed using a clean slide cover (Fadakar et al., 2020). Three DNA samples were prepared in this way. The DNA samples were stored at −20°C until used in polymerase chain reaction (PCR). The PCR to amplify the partial SSU and D2-D3 expansion segments of LSU rDNA was carried out in a total volume of 35 μl (12.1 μl distilled water, 17.5 μl Taq 2X PCR Master Mix, AMPLIQON, Denmark, 1.2 μl of each primers and 3 μl DNA template). The partial SSU rDNA was amplified using the forward primer F22 (5’–TCCAA GGAAGGCAGCAGGC–3’) (Dorris et al., 2002) and reverse primer 1,573 R (5’–TACAAAGGGCAGGGACGTAAT–3’) (Mullin et al., 2005). Primers used for the D2–D3 expansion segments of LSU rDNA amplification were forward primer KK28S-1D1F (5’–AAGGATTCCCTTAGTAACGGCGAGTG–3’) (Kiontke et al., 2004), reverse primer D3B (5’–TCGGAAGGAACCAGCTACTA–3’) (Nunn, 1992). The thermal cycling program for amplifying was as follows: denaturation at 95°C for 4 min, followed by 35 cycles of denaturation at 94°C for 30 s, annealing at 52°C for 40 s, and extension at 72°C for 80 s. A final extension was performed at 72°C for 10 min. To check the successful amplification, 2 μl of the PCR product was loaded on agarose gel including DNA Green Viewer™. The PCR products were purified and sequenced by the Macrogen Macrogen Corporation, South Korea.

The newly generated DNA sequences of the *Oscheius myriophilus* were compared with those of other nematode species available in GenBank using BLAST. The maximal number of sequences of *Oscheius* spp. (Abolafia and Peña-Santiago, 2019) and sequences from several other classic rhabditid genera were selected for reconstructing of the phylogenetic trees. The alignment of SSU sequences was done using the Q-INS‐i algorithm of online version of MAFFT version 7 (http://mafft.cbrc.jp/ alignment/server/) (Katoh and Standley, 2013). To eliminate the ambiguously aligned parts, the online version of Gblocks 0.91b with all the three less stringent parameters was used (http://molevol.cmima.csic.es/castresana/Gblocks_server.html). The alignment of LSU rDNA D2-D3 sequences was done using ClustalW and the alignment was manually edited using MEGA 6 (Thompson et al., 1994). The model of base substitution was selected using MrModeltest 2 (Nylander, 2004). The Bayesian analysis was performed using MrBayes 3.1.2 (Ronquist and Huelsenbeck, 2003) under the GTR + I + G model for five million generations for both SSU and LSU datasets. The Markov chain Monte Carlo (MCMC) chains were sampled every 100 generations for estimating the posterior probabilities (PP) of the phylogenetic trees (Larget and Simon, 1999) using the 50% majority rule. The burn-in phase was set at 25% of the converged runs. Tracer v1.5 software (Rambaut and Drummond, 2009) was used to visualize the results of each run in order to check the effective sample size of each parameter. The outgroup sequences in both phylogenies were selected according to Abolafia and Peña-Santiago (2019). The output file of the used phylogenetic program was visualized using Dendroscope V.3.2.8 (Huson and Scornavacca, 2012) and the tree was redrawin in CorelDRAW software version 2017.

### Pathogenicity of the Oscheius myriophilus against Cydalima perspectalis, and Hyphantria cunea larvae

Different larval stages of BTM were collected from box trees (*Buxus hyrcana* Pojark) in Kishdibi, Talesh, Gilan province, north Iran (37°48′N, 48°53′E) in April–July 2019. The larvae were kept in cages (20×30×30 cm) with holes covered by fine mesh at growth chambers (24°C, 60% R. H., 16:8 h L:D). Fresh leaves of box trees were provided daily to the larvae. Different larval stages, eggs and pupae of FWW were collected from white mulberry (*Morus alba* L.), Caucasian wingnut (*Pterocarya Fraxinifolia* (Lam.)), alder (*Alnus* sp.), and elm (*Ulmus* sp.) trees in Gisum forest Park, Gilan province, north Iran (37°38′N, 49°02′E), during May–July 2019. They were reared on mulberry leaves under laboratory conditions at growth chambers (24°C, 60% R.H., 16:8 h L:D) in plastic containers. Mulberry leaves were changed daily, not allowing to dry completely. The susceptibilities of fourth and fifth instar larvae of BTM and FWW to the *Oscheius myriophilus* were evaluated under laboratory conditions. The larvae were exposed to dauer stage of the nematode in 24-well plates (SPL Life Sciences Co., Ltd. Korea) having a small hole in the lid, including a piece of Whatman filter paper and small pieces of fresh washed leaves. A single larva of insect pests was placed in each well, and inoculated with the infective juveniles of the nematode. Each larva in the treatment was inoculated with 0, 10, 25, 50, 100, 200, 300, and 500 IJs per 1 ml of distilled water. The control larvae received 100 μl of distilled water only. Each treatment was performed in 20 wells with five replicates, therefore, 100 wells were used for each treatment, and a total of 800 larvae were tested for each larval instar of both pests. The plates were placed in plastic containers in a dark growth chamber at 25 ± 2°C, 60% RH, and 16:8 h L:D period. Mortality of the larvae and typical signs of infection including color change of larval body were registered daily during 144 h. Dead treated larval individuals were moved into the white trap to validate the mortality agent was nematode infection. Mortality curves (logrank Mantel–Cox test) of laboratory experiments were analyzed with the Kaplan–Meier method using GraphPad Prism (version 6.07, GraphPad Software, Inc.). The LC50 and LC90 values of EPNs were estimated by probit analysis using SPSS Statistics 17.

## Results

**Figure 1:**
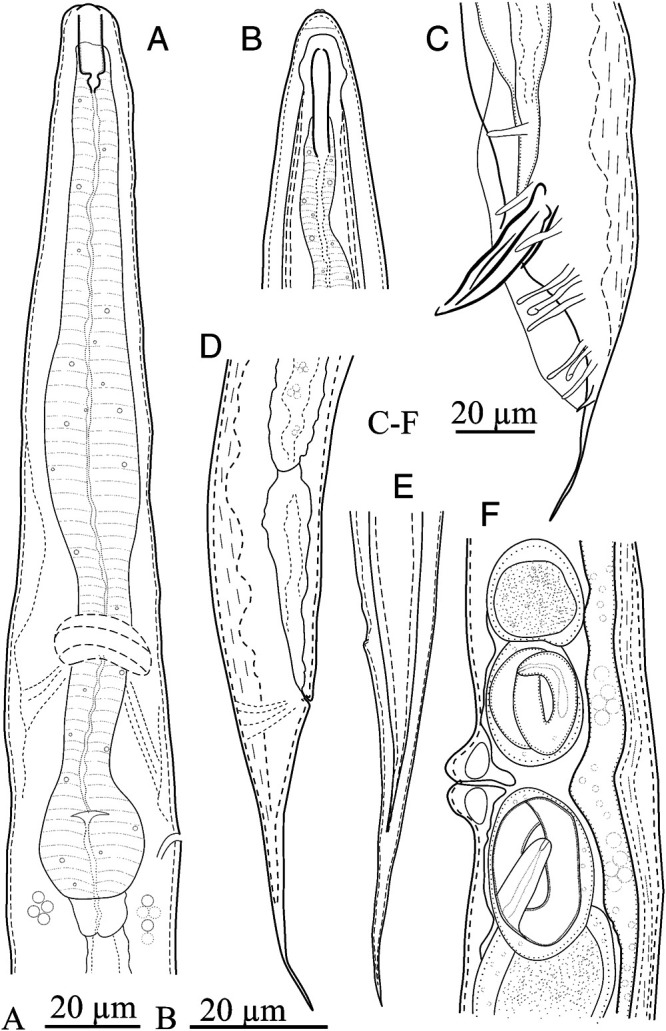
Iranian population of *Oscheius myriophilus* (Poinar, 1986). (A) Pharyngeal region of hermaphrodite; (B: Anterior body region of infective juvenile; (C) Male tail, genital papillae, spicules, and gubernaculum; (D) Posterior body region of hermaphrodite; (E) Infective juvenile tail; (F) Vulval region.

**Figure 2:**
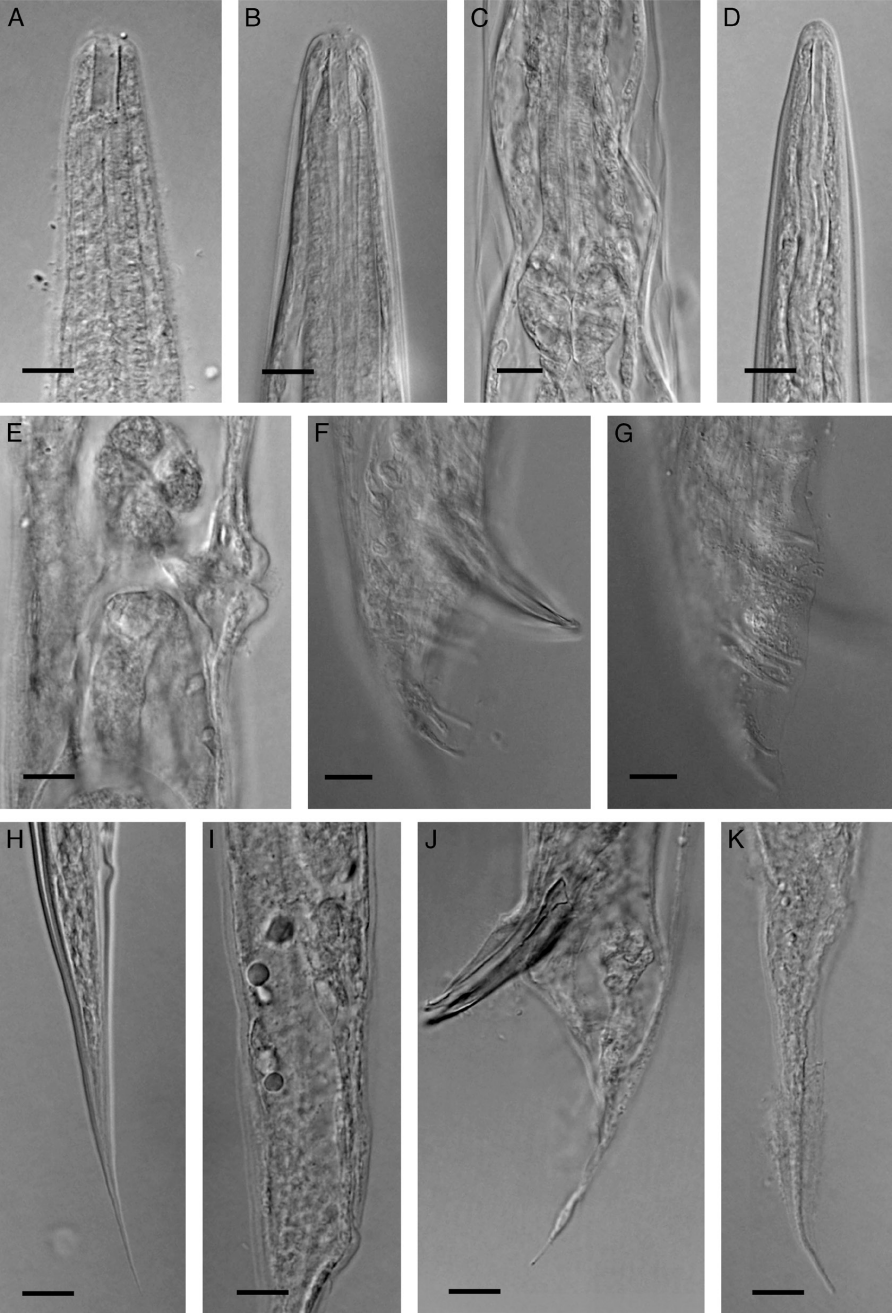
Iranian population of *Oscheius myriophilus* (Poinar, 1986). (A) Anterior body region of hermaphrodite; (B) Anterior body region of male; (C) Part of pharynx (male), (D) Anterior body region of infective juvenile; (E) Vulval region; (F, G) Male tail and genital papillae; (H) Infective juvenile tail; (I) Female rectum; (J) Spicule and tail tip; (K) Hermaphrodite tail (scale bars = 10 µm).

*Oscheius myriophilus* was isolated using soil baiting method in present study. No other EPNs or freeliving bacteriovorus nematodes were isolated using this method in this trial. The brief description of the isolated Iranian population of *Oscheius myriophilus* is given in below. The line drawing and light microphotographs of this population is given in Figs 1 and 2.

### Measurements

Measurements of this population are given in Table 1.

### Hermaphrodite

Small nematodes. Body slender, slightly narrowing towards both extremities, slightly ventrally curved after fixation. Cuticle with very fine transverse striation. Lateral field with four incisures. The labial region continuous with body contour with six outstanding lips. The labial and cephalic sensilla barely visible. Amphidial openings small ellipsoid pores on lateral lips. Stoma rhabditoid, tubular, 4.0 (3.7–4.5) times longer than wide, the cheilorhabdion not strongly cuticularized, metarhabdions bearing minute tubercles and pharyngeal collar present. Pharynx rhabditioid, corpus subcylindrical, muscular, 2.0 to 2.8 times longer than isthmus, with swollen metacorpus, isthmus narrower, basal bulb swollen, the valvular apparatus well-developed. Cardia distinct. Intestine simple, rectum and anus functional, the former longer than anal body width. Excretory pore at the level with the basal bulb. Nerve ring surrounding the isthmus at middle. Hemizonid not clearly seen. Deirids inconspicuous. Reproductive system didelphic-amphidelphic, each gonad reflexed, including mature eggs with juveniles. Vulval lips protruding. Tail elongate-conoid with pointed tip, 1.1 to 1.8 times the rectum length. Phasmids not clearly seen.

### Male

Body straight after fixation. Cuticle smooth, with fine striations. Anterior body region similar to that of female. Excretory pore conspicuous, at the level of basal bulb. Nerve ring surrounding mid-part of isthmus. Hemizonid not observed. Testis monarchic, reflexed ventrally in tip (the reflexed part 150–210 μm). Bursa leptoderan. Nine pairs of genital papillae arranged as 1+1+1/3+3, G5 and G8 opening dorsally (Abolafia and Peña-Santiago, 2019). Spicules paired, separate, symmetrical and slightly curved, their head (manubrium) triangular and distal tip finely rounded, shaft (calomus) short, blade (lamina) with two internal ribs. Gubernaculum ventrally flattened, about 45.1% (41.6–48.8) of the spicule length, long. Tail conoid, ending to a spike-like differentiation.

### Infective larvae

Straight when heat killed, the sheath conspicuous. Labial papillae inconspicuous. Stoma tubular, about 4.25 to 6.8 times longer than wide. Reproductive system not developed. Tail elongate conoid.

### Remarks

Iranian population of *O. myriophilus* was recovered from *G. mellonella* larvae in laboratory culture during present study. It fits well with the type population described by Poinar (1986) recovered from *Oxidis gracilis* (Koch). However, some morphometric variations were observed. The body length of dauer larvae of Iranian population were slightly longer (583–791 vs 504–611 μm), and they had posteriorly located excretory pore (110–129 vs 97–114 μm from anterior end) and longer tail (85–100 vs 75–80 μm). Compared to the Turkish population reported by Erbaş et al. (2017), the aforementioned indices were closer (see Table 1).

The hermaphrodites of Iranian population had slightly shorter body (870–1,247 vs 1,200–1,500 μm), and slightly anteriorly located excretory pore (135–167 vs 165–190 μm distance from anterior end) in comparison with the type population (Poinar, 1985). However, the body length of hermaphrodites of Iranian population are closer to the body length of the hermaphrodites of Australian population studied by Sudhaus and Schulte (1989). According to Sudhaus and Schulte (1989), the c ratio of the type hermaphrodites (2.2–3.5) should be corrected to 8.9–13.0. The range for this index in Iranian population was 8.1–10.9.

### Molecular and phylogenetic analyses

**Figure 3:**
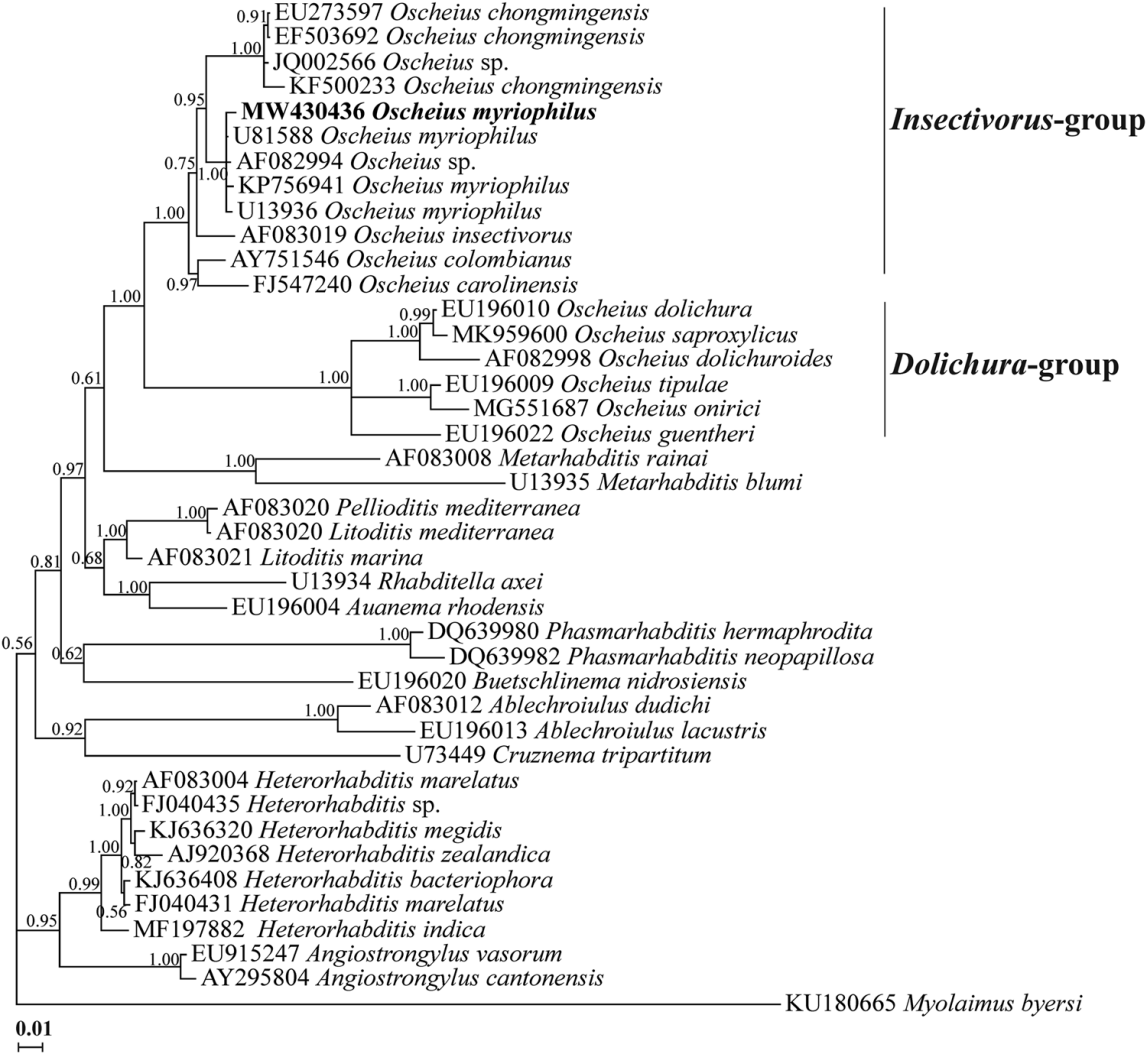
Bayesian 50% majority rule consensus tree inferred using partial SSU rDNA sequence of Iranian population of *Oscheius myriophilus* (Poinar, 1986) under the GTR + G + I model. Bayesian posterior probability values more than 0.50 are given for appropriate clades. The Iranian population is indicated in bold.

For the molecular phylogenetic studies of the Iranian population of *Oscheius myriophilus*, a partial fragment of SSU rDNA (937 nt long, accession number MW430436) and two sequences of LSU rDNA D2-D3 (740 and 814 nt long, accession numbers MT897258 and MT897259) were used. The BLAST search using the SSU sequence revealed it has 99.57–99.89% identity with three isolates of *O. myriophilus* (U81588, U13936, KP756941), and 99.74% identity with one unidentified isolate of the genus (AF082994) probably belonging to *O. myriophilus*. Fig. 3 shows the SSU phylogenetic tree reconstructed using 40 ingroup, and one outgroup sequences (for species names and accession numbers see the tree). The Iranian isolate of *O. myriophilus* has fell into the maximally supported clade including four aforementioned sequences (U81588, U13936, KP756941, AF082994). One gap, and one to four indels were observed between the aforementioned sequences (including the SSU of the Iranian isolate) while aligning.

**Figure 4:**
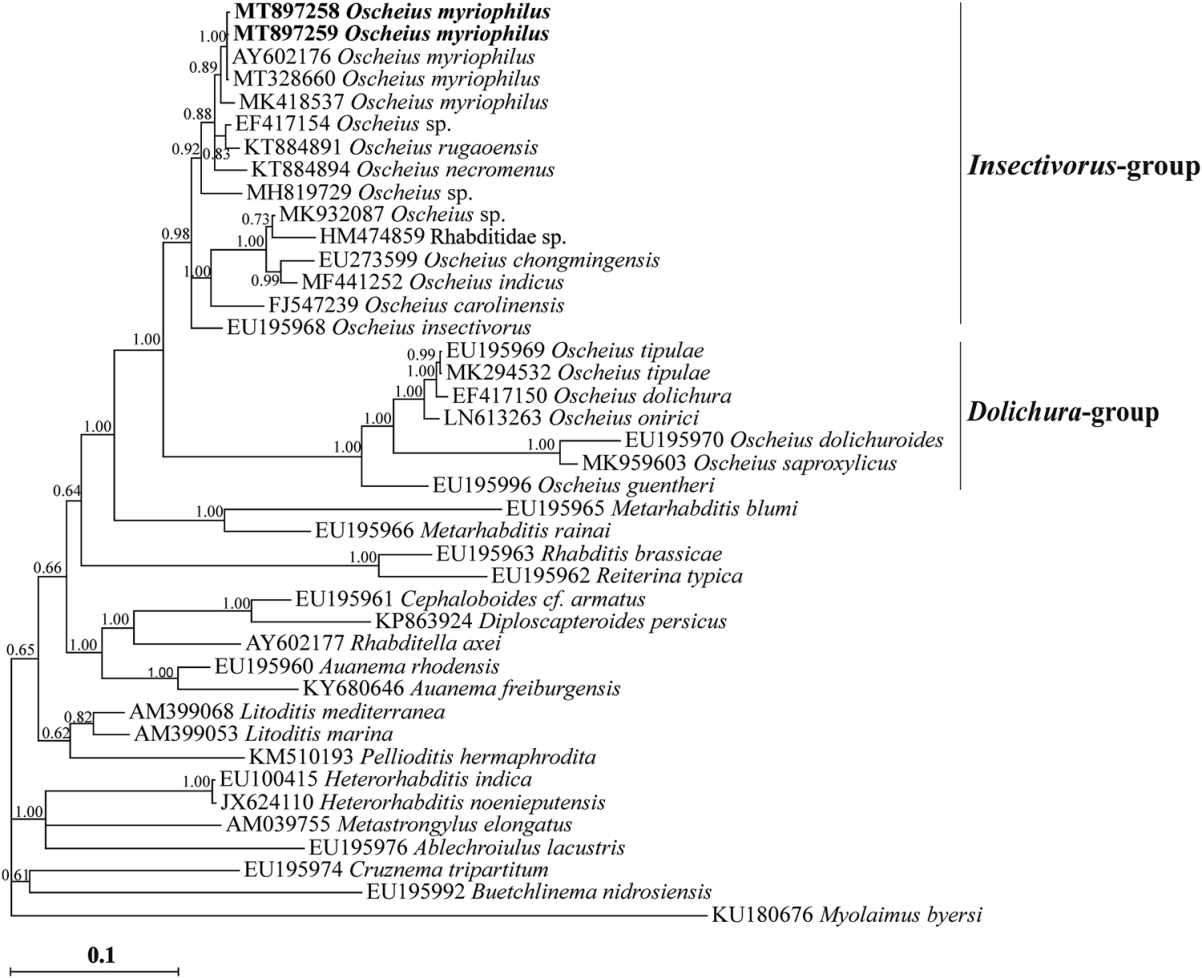
Bayesian 50% majority rule consensus tree inferred using the partial LSU rDNA sequences of Iranian population of *Oscheius myriophilus* (Poinar, 1986) under the GTR + G + I model. Bayesian posterior probability values more than 0.50 are given for appropriate clades. The sequences of Iranian population are indicated in bold.

The two LSU D2-D3 sequences of the Iranian population of *Oscheius myriophilus* were identical while aligning. Their Blast search revealed they are almost identical to two LSU sequences of two isolates already deposited into the GenBank database (AY602176, MT328660) and have maximal (100%) identity with several other isolates of the species (e.g. MN389700-MN389728). Fig. 4 represents the Bayesian phylogenetic tree inferred using the 40 LSU sequences of ingroup and one outgroup taxa (for species names and accession numbers see LSU tree). In this tree, the two LSU sequences of Iranian isolate of the species have fell into a clade including two aforementioned sequences; and the isolate identified under the species name *O. myriophilus* (MK418537) is the sister clade to them.

### Pathogenicity of Oscheius myriophilus against Cydalima perspectalis and Hyphantria cunea larvae

No bacterial or fungal infection of dead larvae of two insect pests was observed in pathogenicity tests. *Oscheius myriophilus* caused 100% mortality on the fifth instar larvae (Log-rank (Mantel–Cox) test, *χ* ^2^=44.90, df=1, *P*<0.0001 compared with control); and 90% mortality (Log-rank (Mantel–Cox) test, *χ* ^2^=32.22, df=1, *P*<0.0001) on the fourth instar larvae of BTM at 500 IJs/ml 48 h after nematode inoculation. When the concentration of nematodes decreased from 500 to 300 IJs/ml, mortality of BTM were also decreased from 100% to 97% (Log-rank (Mantel–Cox) test, *χ* ^2^=34.81, df=1, *P*<0.0001) for fifth instar larvae, and from 90 to 77% (Log-rank (Mantel–Cox) test, *χ* ^2^=21.60, df=1, *P*<0.0001) for fourth instar larvae at 48 h of exposure.

In the bioassay against FWW larvae, *Oscheius myriophilus* caused 95% mortality on fifth instar larvae (Log-rank test, *χ* ^2^

=32.67, df=1, *P*<0.0001) at the dose of 500 IJs/ml within two days after exposure, and 83% on the fourth instar larvae (Log-rank test, *χ* ^2^=23.79, df=1, *P*<0.0001) in the same concentration and time. With decreasing the nematode concentration to 300 IJs/ml, a 81% mortality in fifth instar larvae of FWW (Log-rank test, *χ* ^2^=21.87, df=1, *P*<0.0001) and 76% mortality in fourth instar larvae of FWW (Log-rank test, *χ* ^2^=16.55, df=1, *P*<0.0001) was observed after 48 h of treatment. Low levels of mortality (32–83%) were recorded whit 50 IJs/ml dosage of *O. myriophilus* on fifth larval instars of FWW (Log-rank test, *χ* ^2^=7.93, df=1, *P*=0.004) and a 22–65% mortality was observed when 50 IJs/ml dosage of *O. myriophilus* were applied to fourth instar larvae of FWW (Log-rank test, *χ* ^2^=4.33, df=1, *P*=0.03) for 24–144 h. No significant differences were observed in FWW larval death rate compared with the controls in 10 IJs/ml dosage of nematode on both larval stages.

Table 2 shows the LC50 and LC90 values at 48 h post-treatment of the nematode. The lowest LC50 value (74.5) was obtained on fifth instar larvae of BTM, while the highest value (197.3) was for fourth instar larvae of FWW.

**Table 2.** Comparative LC50 and LC90 values of *Oscheius myriophilus* (Poinar, 1986) for *Cydalima perspectalis* (Walker, 1859) and *Hyphantria cunea* (Drury, 1773) at 48 h post-treatment by EPNs

Host	Organism	*χ* ^2^ (df=6)	*P*-value	Intercept ± SE	Slope ± SE	LC50	LC90
*C. perspectalis* (fourth larval instars)	*O. myriophilus*	66.70	0.000	−0.79 ± 0.06	0.005 ± 0.000	152.70 (68.1–267.1)	400.30 (280.2–802.7)
*C. perspectalis* (fifth larval instars)	*O. myriophilus*	72.18	0.000	−0.68 ± 0.06	0.005 ± 0.00	74.53 (20.70–133.71)	217.02 (150.92–438.99)
*H. cunea* (fourth larval instars)	*O. myriophilus*	65.24	0.000	−0.92 ± 0.06	0.005 ± 0.000	197.30 (110.0–339.1)	470.90 (332.3–934.9)
*H. cunea* (fifth larval instars)	*O. myriophilus*	78.00	0.000	−0.52 ± 0.06	0.005 ± 0.000	99.94 (−20.4–211.27)	346.47 (227.4–906.9)

## Discussion

The genus *Oscheius* was established by Andrássy (1976). It is mainly characterized by a short buccal tube about as long as wide, and absence of median pharyngeal swelling. Abolafia and Peña-Santiago (2019) presented an excellent revision on taxonomy and phylogeny of the genus, discussed on its valid subgenera and intragenus grouping of the valid species. After the SSU and LSU phylogenetic trees in their study, the genus seems to be monophyletic based on both markers.

As described in introduction, there are two intragenus groups under the genus. *Oscheius myriophilus* belongs to the insectivorous group because of its lips, posterior body region and spicules features. It was originally isolated from the gut lumen, malpighian tubes and haemocoel of the garden millipede, *Oxidis gracilis*, in California (Poinar, 1986), and was later recovered in Australia (Sudhaus and Schulte, 1989), Turkey (Erbaş et al., 2017), and Mexico (Castro-Ortega et al., 2020).

During the present study, it was recovered from natural forests of north Iran. This isolate showed some morphological variations compared to the type population as already discussed, but compared to other isolates, no remarkable differences were observed (see Table 1).

The type population of *Oscheius myriophilus* is described solely based upon traditional criteria, and molecular data are not available for it. On the other hand, there are currently other SSU sequences assigned to the species, and it seems the sequence AF082994 does also belong to this species. No morphological data are available for the sequences. No remarkable differences were observed between the SSU sequences of the Iranian isolate and previously deposited sequences of the species into the GenBank. The LSU sequences of the present population were also identical to several LSU sequences already deposited into the GenBank database, as already discussed. Compared to the other LSU sequence assigned to *O. myriophilus* (MK418537), the newly generated LSU sequences had remarkable differences (2 gaps and 13 indels), thus the identity of this sequence needs further validations. The presently resolved SSU phylogeny, is similar to the resolved tree by Abolafia and Peña-Santiago (2019), corroborating members of the Insectivorus-group and Dolichura-group form monophyletic groups, both of which forming the monophyletic genus *Oscheius* (based on currently available data). The similar pattern was also observed in LSU phylogeny.

The virulence of *Oscheius myriophilus* against *Galleria mellonella* larvae was investigated by Castro-Ortega et al. (2020) and the LC50 value of 4.732 was calculated. According to them, *Oscheius myriophilus* has the potential to be used as a biological control agent. The results of the current study revealed that *O. myriophilus* has remarkable efficacy against BTM and FWW larvae in the laboratory condition. It showed more virulence against BTM larvae than FWW; and a 500 IJs/ml dose of *O. myriophilus* resulted in the highest mortality (83–100%) on fifth larval instars of BTM in 24–144 h post treatments. This result highlights the potential of using of this entomopathogenic nematode in controlling programs of this pest. Previously, Ye et al. (2010) showed the virulence of *O. carolinensis* Ye et al., 2010 against third instar of *Pieris rapae* (L.); and reported a 100% larval mortality in less than 48 h after inoculation. In the study of Zhang et al. (2012), *O. rugaoensis* had high virulence on *G. mellonella,* causing a 95% larval mortality at the nematode concentration of 284 (188.1–1,022.6) IJs/ml. The LC50 of the nematode on *G. mellonella* within 48 h after infection was 24.35 (10.3–53.6) IJs/ml. In the study by Torrini et al. (2015), *O. onirici* infected 46% of *G. mellonella*, and 58% of *Tenebrio molitor* larvae, during one week in laboratory condition. Zhou et al. (2017) recorded a 93.1% larval mortality of *G. mellonella* at 320 IJs/ml after 48 h of inoculation of *O. microvilli* in the laboratory conditions. The LC50 of the *O. microvilli* against *G. mellonella* within 48 h of infection was 69.1 (41.9–111.9) dauer juveniles per milliliter.

**Figure 5:**
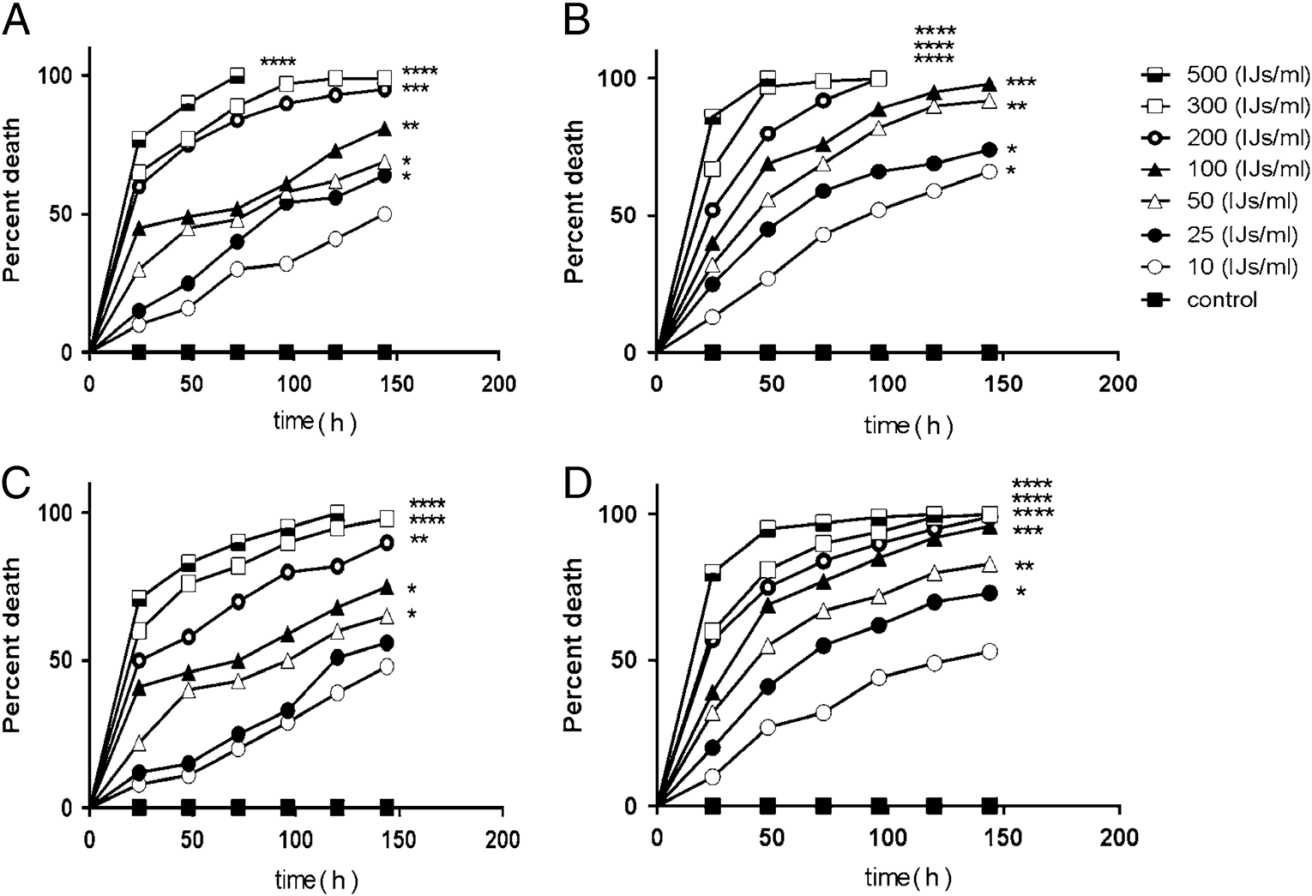
Death of *Cydalima perspectalis* (Walker, 1859) and *Hyphantria cunea* (Drury, 1773) larvae after exposure to different doses of *Oscheius myriophilus* (Poinar, 1986) in 24–144 h post treatment in laboratory bioassays. (A) fourth instar larvae of *Cydalima perspectalis* exposed to *Oscheius myriophilus* (B) fifth instar larvae of *Cydalima perspectalis* exposed to nematode (C) fourth instar larvae of *H. cunea* exposed to nematode (D) fifth instar larvae of *Hyphantria cunea* exposed to nematode, Log-rank test (Mantel–Cox) on GraphPad Prism software was performed to determine statistical significance for the death curves. *p< 0.05, **p<0.01, ***p<0.001, ****p<0.0001, compared with control.

The Hyrcanian forests in north Iran has high annual rainfall, fertile soils, humid temperate, and high growth capacity (Marvie Mohajer, 2004). The flora of the region are of high importance and are national genetic resources, could be at risk of several pathogens and pests. The natural pathogens of pests have long been known to play a major role in the population dynamics of many important forest pests (Bonsall, 2004). So far, only few species of EPNs have been recorded from Iran and very few surveys have evaluated their applicability in forest pests controlling programs. The observed results in present study opens a new field for further studies to use the potential of these natural biocontrol agents in large scales in natural forests. The use of EPNs can be an appropriate alternative to chemical compounds in forestry, and this study can serve as a basis for future research to develop effective application technology, mass-production, and formulation technology of EPNs (Fig. 5).
